# Treatment delay and fatal outcomes of pulmonary tuberculosis in advanced age: a retrospective nationwide cohort study

**DOI:** 10.1186/s12879-017-2554-y

**Published:** 2017-06-24

**Authors:** Chih-Hsin Lee, Jann-Yuan Wang, Hsien-Chun Lin, Pai-Yang Lin, Jer-Hwa Chang, Chi-Won Suk, Li-Na Lee, Chou-Chin Lan, Kuan-Jen Bai

**Affiliations:** 1 Division of Pulmonary Medicine, Department of Internal Medicine, Wanfang Hospital, Taipei Medical University, No. 111, Sec. 3, Hsing-Long Rd., Taipei, 116 Taiwan; 2 Division of Pulmonary Medicine, Department of Internal Medicine, School of Medicine, College of Medicine, Taipei Medical University, No. 250, Wuxing St., Taipei, 110 Taiwan; 3 Department of Internal Medicine, National Taiwan University Hospital, No.7, Chung Shan S. Rd., Taipei, 100 Taiwan; 4 School of Respiratory Therapy, College of Medicine, Taipei Medical University, No. 250, Wuxing St., Taipei, 110 Taiwan; 5 Department of Laboratory Medicine, National Taiwan University Hospital, No.7, Chung Shan S. Rd., Taipei, 100 Taiwan; 6 Division of Pulmonary Medicine, Taipei Tzu Chi Hospital, Buddhist Tzu Chi Medical Foundation, No. 289, Jianguo Rd., Xindian Dist, New Taipei, 231 Taiwan; 7 School of Medicine, Tzu Chi University, No.701, Sec. 3, Zhongyang Rd., Hualien, 970 Taiwan

**Keywords:** Tuberculosis, Infection and inflammation, Clinical respiratory medicine, Clinical epidemiology

## Abstract

**Background and objective:**

Studies focusing on pulmonary tuberculosis in advanced age (≥80 years) are lacking. This study aimed to explore treatment delay, outcomes and their predictors in this group.

**Methods:**

Adult (≥20 years) patients with pulmonary tuberculosis were identified from the National Health Insurance Research Database of Taiwan from 2004 to 2009. Treatment completion and mortality rates were noted at one year after treatment.

**Results:**

Among the 81,081 patients with pulmonary tuberculosis identified, 13,923 (17.2%) were aged ≥80 years, and 26,897 (33.2%) were aged 65–79 years. The treatment completion, mortality rates and treatment delay were 54.8%, 34.7% and 61 (12–128) [median, (1st-3rd quartiles)] days in patients aged ≥80 years, 68.3%, 18.5% and 53 (8–122) days in patients aged 65–79 years, and 78.9%, 6.5% and 21 (1–84) days in patients aged <65 years, respectively. The elder patients were more likely to receive second-line anti-tuberculosis agents. The treatment completion rate decreased with older age, female sex, comorbidities, low income, requiring second-line anti-tuberculosis agents, severity of pulmonary tuberculosis and longer treatment delay. Older age, female sex, comorbidities, low income, and not undergoing rapid molecular diagnostic tests were independently associated with longer treatment delays.

**Conclusions:**

Pulmonary tuberculosis in advanced age has a longer treatment delay and a higher mortality rate. Applying rapid molecular diagnostic tools may reduce treatment delay and should be integrated into the diagnostic algorithm for pulmonary tuberculosis, particularly in elderly patients.

**Electronic supplementary material:**

The online version of this article (doi:10.1186/s12879-017-2554-y) contains supplementary material, which is available to authorized users.

## Background

Because of increasing life expectancy and declining birth rates, the ageing population problem has become a critical worldwide public health concern, particularly in developed countries [1]. Dysfunction in cellular immunity caused by chronic comorbidities, malnutrition, and age-related changes can render elderly people more susceptible to infectious agents, such as *Mycobacterium tuberculosis* [2, 3]. In 2015, 10.4 million people were diagnosed as active tuberculosis (TB) and among them, 1.8 million died [4]. In industrialised societies, the trend of institutionalised care further exposes elderly patients to a higher risk of TB infection. The elderly population therefore represents a large reservoir of TB infection. In developing countries, TB continues to affect all susceptible individuals, including elderly adults [5].

Delay in initiation of anti-TB treatment is a major impediment to effective control of TB [6]. However, in elderly people, the clinical presentations of TB can be myriad and easily confused with other age-related illnesses [7]. Although the standard four-combined anti-TB treatment is highly effective, it is associated with a high pill burden, long treatment course, and severe drug-related adverse events. Elderly patients are more prone to experience adverse events such as severe hepatotoxicity during anti-TB treatment [8]. These factors result in a unique challenge and suboptimal outcomes in management of TB among the geriatric population.

Sputum smear microscopy remains the most common method for diagnosing pulmonary TB (PTB), but smear-positive TB accounted for only 56% of all notified new TB cases [4]. A mycobacterial culture, although more sensitive, requires an average of 9.7 and 20.2 days to detect *M. tuberculosis* in liquid and solid culture media, respectively [9]. The advent of rapid molecular diagnostic tools, which are sensitive, specific, and quick, provides new opportunities to facilitate the microbiological diagnosis of PTB [10, 11].

In this nationwide retrospective cohort study, we investigated the impact of advanced age (≥80) on delay and outcome of anti-TB treatment with an emphasis on the influence of rapid molecular diagnostic tools.

## Methods

The National Health Insurance (NHI) programme of Taiwan is a compulsory insurance system covering 99.6% of the national population with a benefit package including comprehensive inpatient and outpatient medical services. The claims data were collected systemically and de-identified before being released for research purposes. The data were issued by the National Health Research Institute with delegation of authority from the Ministry of Health and Welfare under license for the current study.

In this study, patients with PTB during 2004–2009 were selected from the NHI Research Database (NHIRD) and followed-up until death or 31st December 2010, whichever came first. The Institutional Review Board of National Taiwan University Hospital approved the study (NTUH REC: 201,309,064 W).

### Selection criteria for pulmonary tuberculosis

PTB was defined as having at least two outpatient visits or any inpatient record with compatible diagnoses of PTB (International Classification of Diseases, Ninth Revision, Clinical Modification [ICD-9-CM] code 010–012, 018) [12, 13]. Participants needed to have been prescribed at least two anti-TB drugs simultaneously for ≥120 days within a period of 180 days as well as at least one prescription of ≥3 anti-TB drugs. Patients were also considered to have PTB if they had a positive TB culture or received ≥2 anti-TB drugs simultaneously for ≥30 days during the last 3 months before loss to follow-up [13]. Patients who were diagnosed with non-tuberculous mycobacterial infection (ICD-9-CM code 031) during the last 2 months of anti-TB treatment were excluded. The annual number of PTB cases identified with abovementioned criteria has been verified with that reported from the Taiwan Centers for Disease Control [12, 13].

### Treatment outcomes of pulmonary tuberculosis

The index date was defined as the date when anti-TB treatment began. For those who did not receive anti-TB treatment, the index date was defined as the date of death. The first-line anti-TB agents included isoniazid, rifampicin/rifabutin, ethambutol and pyrazinamide. The second-line anti-TB agents included quinolones, aminoglycosides, prothionamide, cycloserine, terizidone and para-aminosalicyclic acid. Treatment outcome was recorded 1 year after the index date. Anti-TB treatment was traced until the last prescription comprising two or more anti-TB drugs followed by no further anti-TB agents in the subsequent 60 days. Anti-TB treatment was considered completed for those who remained alive at the end of anti-TB treatment and received ≥144 days of rifamycin (a corresponding adherence ≥80% of 180 days) and a total treatment duration ≤365 days. Mortality was recorded if death occurred within 365 days and before the anti-TB treatment was completed [14].

### Anti-tuberculosis treatment delay

Treatment delay was calculated as the interval from the earliest date fulfilling any two events possibly indicating the onset of PTB to the index date (Additional file 1: Figure S1). Events possibly indicating the onset of PTB included: diagnoses of TB or pneumonia (ICD-9-CM code 480–486 or 507), consulting pulmonologists or infectious disease specialists, receiving chest radiography, taking airway medications or antibiotics, and requiring a mycobacterial culture or *M. tuberculosis*–nucleic acid amplification test (MTB–NAAT) within 6 months prior to the index date. Airway medications included oral antitussives, mucolytic agents, and sympathomimetics. Antibiotics included penicillins, cephalosporins, quinolones, carbapenems, and macrolides. The treatment delay was further decomposed into two parts. Delay in arousing clinical suspicion was defined as delay prior to the date first mycobacterial culture study was prescribed. Delay due to technical limitation in diagnosis was the interval from first mycobacterial culture study to the start of anti-TB treatment.

### Possible confounding factors

Underlying comorbidities that have been shown to interfere with the treatment outcomes and delays were recorded at the index date [12, 13, 15]. The low-income group was identified from the insurance status and required the annual household income to be below 4500 US dollars [16].

Baseline TB severity was assessed by the presence of extra-pulmonary TB (diagnostic code of ICD-9-CM codes 012.0, 013 ~ 018), requiring second-line anti-TB agents and the requirement of hospitalization, intensive care unit admission, invasive and non-invasive mechanical ventilatory support during the first 14 days of anti-TB treatment [15].

The healthcare system factors of initial medical visits indicating PTB onset, including hospital accreditation level, specialty and location, were recorded. The location was classified as an urban (population density ≥ 1500 people/km^2^) or rural area.

### Statistical analysis

Data are expressed as either the median (first to third quartiles) or number (%). Intergroup differences were compared using the Mann–Whitney U test for numerical variables and the chi-square test or Fisher’s exact test, as appropriate, for categorical variables. Multivariate logistic regression analysis, including age, sex, comorbidities, low-income status, baseline TB severity, healthcare system factors, and treatment delay, was applied to identify the independent predictors of anti-TB treatment completion within 1 year. Factors influencing the length of treatment delay were evaluated using multivariate linear regression analysis. A two-sided *p* value <0.05 was considered significant. All analyses were performed using SAS software (Version 9.2, SAS Institute Inc., Cary, NC, USA).

### Subpopulation and sensitivity analyses

Subpopulation analyses were performed to investigate the impact of the MTB–NAAT on treatment delay in three subgroups: (i) patients with age ≥ 65 years; (ii) patients with age ≥ 80 years; and (iii) patients whose delay due to technical limitation longer than 7 days, implying that they were smear-negative PTB cases.

A sensitivity analysis was performed by adopting a stricter definition for treatment delay, which was calculated as the interval between the earliest date fulfilling any three events possibly indicating the onset of PTB and the index date.

## Results

**Fig. 1:**
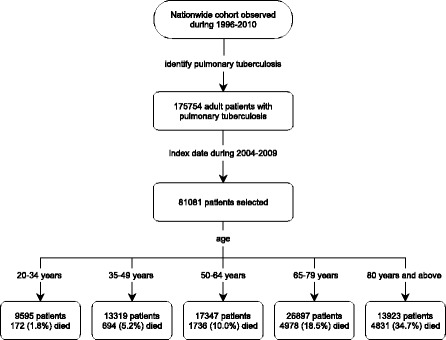
Flow chart of case selection from the National Health Insurance Research Database of Taiwan, with outcome recorded at 1 year after the index date

**Fig. 2:**
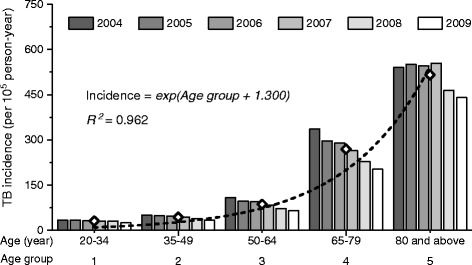
Incidence of pulmonary tuberculosis in Taiwan among different age groups from 2004 to 2009

From the nationwide database, 81,081 adult patients with PTB were identified (Fig. 1). Among them, 3747 (4.6%) died before anti-TB treatment began. The median age was 65.2 (47.5–76.9) years, with a male–female ratio of 2.25. The incidence rate of PTB was exponentially correlated to the age (*R* ^2^ = 0.962; Fig. 2).

The clinical characteristics and treatment courses are summarised in Table 1. More PTB cases were ≥80 years after 2006 than those before, reflecting the trend of ageing. The most common underlying comorbidities were diabetes mellitus (28.4%), malignancy (8.7%), and chronic obstructive pulmonary disease (8.2%). Compared with patients 65–79 years of age, patients with age ≥ 80 years had generally lower prevalences of comorbidities except for chronic obstructive pulmonary disease. Extra-pulmonary tuberculosis was more common among younger patients. Computerised tomography (CT) scan was performed more frequently among older patients (age of 65 years or more). Invasive diagnostic procedures such as bronchoscopy and CT-guided biopsy were done more frequently among patients 65–79 years of age and less common among patients with age ≥ 80 years.

**Table 1:** Clinical characteristics of the 81,081 adult patients with pulmonary tuberculosis diagnosed from 2004 to 2009

Age (years)	20–64 *N* = 40,261	65–79 *N* = 26,897	80 and above *N* = 13,923
Male	26,377 (65.5%)	19,935 (74.1%)^*^	9855 (70.8%)^*,†^
Pre-DOTS era (2004–2005)	14,694 (36.5%)	10,030 (37.3%)^*^	4189 (30.1%)^*,†^
DOTS era (2006–2009)	25,567 (63.5%)	16,867 (62.7%)^*^	9734 (69.9%)^*,†^
Comorbidities			
Diabetes mellitus	9148 (22.7%)	9606 (35.7%)^*^	4248 (30.5%)^*,†^
COPD	950 (2.4%)	3114 (11.6%)^*^	2568 (18.4%)^*,†^
Malignancy	2242 (5.6%)	3239 (12.0%)^*^	1583 (11.4%)^*,†^
ESRD	786 (2.0%)	882 (3.3%)^*^	241 (1.7%)^†^
Autoimmune disease	435 (1.1%)	306 (1.1%)	78 (0.6%)^*,†^
Liver cirrhosis	316 (0.8%)	99 (0.4%)^*^	35 (0.3%)^*^
Pneumoconiosis	12 (0.0%)	44 (0.2%)^*^	10 (0.1%)^*,†^
AIDS	404 (1.0%)	36 (0.1%)^*^	9 (0.1%)^*^
Transplantation	100 (0.25%)	24 (0.06%)^*^	0^*,†^
Low income status	1657 (4.1%)	838 (3.1%)^*^	373 (2.7%)^*,†^
Diagnostic procedures during the last 2 months before anti-TB treatment			
Bronchoscopy	3857 (9.6%)	3010 (11.2%)^*^	1136 (8.2%)^*,†^
CT scan	13,504 (33.5%)	11,255 (41.8%)^*^	5828 (41.9%)^*^
CT-guided biopsy	702 (1.7%)	546 (2.0%)^*^	156 (1.1%)^*,†^
Healthcare system factors of initial visits			
Hospital accreditation level		*	*^,†^
Medical centers	5525 (13.7%)	3985 (14.8%)	2256 (16.2%)
Regional hospitals	14,657 (36.4%)	10,392 (38.6%)	6545 (47.0%)
Local hospitals or clinics	20,079 (49.9%)	12,520 (46.5%)	5122 (36.8%)
In urban area	30,862 (76.7%)	18,583 (69.1%)^*^	10,199 (73.3%)^*,†^
Pulmonologists or infection specialists	7170 (17.8%)	4255 (15.8%)^*^	2677 (19.2%)^*,†^
Baseline TB severity			
Extrapulmonary involvement	4317 (10.7%)	2531 (9.4%)^*^	846 (6.3%)^*,†^
Second-line anti-TB drugs ≥14 days	5695 (14.1%)	5245 (19.5%)^*^	2951 (21.2%)^*,†^
Within 14 days of commencing anti-TB treatment			
Hospitalisation	17,946 (44.6%)	15,038 (55.9%)^*^	9288 (66.7%)^*,†^
Admission to intensive care units	2337 (5.8%)	3353 (12.5%)^*^	2826 (20.3%)^*,†^
Invasive ventilatory support	1886 (4.7%)	3065 (11.4%)^*^	2797 (20.1%)^*,†^
Non-invasive ventilatory support	290 (0.7%)	533 (2.0%)^*^	478 (3.4%)^*,†^
Duration of anti-TB treatment (day)	212 (185–281)	204 (181–277)^*^	189 (127–260)^*,†^
Treated with isoniazid	188 (152–259)	184 (86–246)^*^	159 (41–209)^*,†^
Treated with rifamycin	191 (171–259)	185 (141–243)^*^	167 (50–210)^*,†^
Treated with ethambutol	176 (144–240)	169 (75–214)^*^	138 (39–189)^*,†^
Treated with pyrazinamide	63 (49–87)	58 (28–81)^*^	49 (7–70)^*,†^
Intensive phase (first 2 months)			
Treated with isoniazid (day)	60 (53–60)	59 (42–60)^*^	53 (19–60)^*,†^
Treated with rifamycin (day)	58 (50–60)	54 (42–60)^*^	49 (26–58)^*,†^
Treated with ethambutol (day)	57 (51–58)	54 (38–58)^*^	49 (21–57)^*,†^
Treated with pyrazinamide (day)	54 (41–60)	47 (19–57)^*^	36 (3–53)^*,†^
Anti-TB treatment outcome at one year			
Completed	31,756 (78.9%)	18,377 (68.3%)^*^	7623 (54.8%)^*,†^
Died	2602 (6.5%)	4978 (18.5%)^*^	4831 (34.7%)^*,†^
Died within 2 months	1145 (2.8%)	1926 (7.2%)^*^	1944 (14.0%)^*,†^

*Abbreviations*: *AIDS* acquired immunodeficiency syndrome, *COPD* chronic obstructive pulmonary disease, *CT* computerised tomography, *DOTS* directly observed treatment, short course, *ESRD* end-stage renal disease

Data are expressed as the median (1st–3rd quartiles) or number (%) as appropriate

*P-value <0.05 compared against the group with age of 20–64 years. ^†^ *P*-value <0.05 compared against the group with age of 65–79 years

**Fig. 3:**
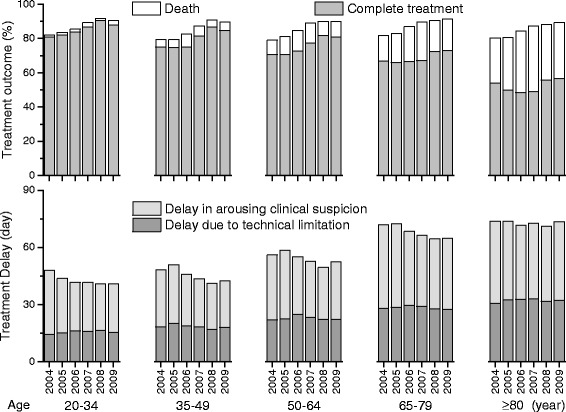
Proportion of patients with complete treatment and fatal outcome (upper panel) and duration of delay in anti-tuberculosis treatment (lower panel) among different age groups

The elder patients required more outpatient visits, emergency room visits, admissions, chest x ray, mycobacterial culture, and MTB-NAAT studies to confirm the TB diagnosis and to start the anti-TB treatment (Additional file 1: Table S3). Among the ageing population, the duration of anti-TB treatment as well as duration covered by each first-line anti-TB drug was shorter while second-line anti-TB drugs were prescribed more frequently; indicating regimen modification. The elder patients had higher baseline disease severity reflected by the higher probabilities of requiring hospitalisation, intensive care, and mechanical ventilatory support during the first 14 days of anti-TB treatment, and consumed more medical resources during the anti-TB treatment course. However, the elder patients carried a lower treatment completion rate and a higher mortality rate (Fig. 3, Table 1). Female patients were younger, had less comorbidities, and a lower mortality rates than male patients (Additional file 1: Table S1).

**Fig. 4:**
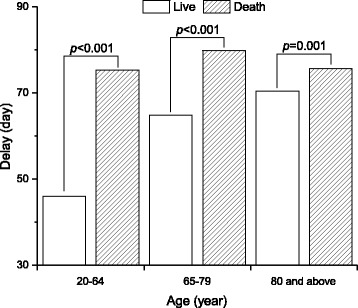
Duration of delay in anti-tuberculosis treatment of patients categorised by treatment outcomes among different age groups

Additional file 1: Table S2 summarises specific events that indicated the PTB onset. The prescription of airway medications or antibiotics tended to occur much earlier than chest x ray examination as well as mycobacterial study in the clinical course. The treatment delay was longer among the elderly than that among younger patients (Fig. 2). A later TB diagnosis year was associated with a shorter treatment delay in all age groups (*P* < 0.001) except for patients ≥80 years old (*P* = 0.678). The treatment delay was longer among patients with a fatal outcome (71 [11–139] days, Fig. 4) than those who completed treatment (33 [4–99] days) (*P* < 0.001)

The overall treatment completion rate within 1 year was 71.2%. Multivariate logistic regression showed an association of lower treatment complete rate with age and longer treatment delay, after adjusting the effect of sex, co-morbidities, healthcare system factors of initial visits, baseline TB severity, and use of second-line anti-TB agents (Table 2).

**Table 2:** Multivariate logistic regression analysis for predictors of complete treatment within 1 year after beginning anti-tuberculosis (TB) treatment

	Number	Completion rate	Unadjusted OR (95% CI)	*P*-value	Adjusted OR (95% CI)	*P*-value
Implementation of DOTS						
Pre-DOTS era (2004–2005)	28,913	68.9%	1		1	
DOTS era (2006–2009)	52,168	72.5%	1.19 (1.16, 1.23)	<0.001	1.25 (1.21, 1.30)	<0.001
Age (years)						<0.001
20–34	9595	85.0%	1		1	
35–49	13,319	79.1%	0.67 (0.63, 0.72)	<0.001	0.84 (0.78, 0.91)	<0.001
50–64	17,347	75.3%	0.54 (0.51, 0.58)	<0.001	0.81 (0.75, 0.87)	<0.001
65–79	26,897	68.3%	0.38 (0.36, 0.41)	<0.001	0.69 (0.64, 0.74)	<0.001
80 and above	13,923	54.8%	0.21 (0.20, 0.23)	<0.001	0.43 (0.40, 0.46)	<0.001
Sex						
Female	24,914	71.7%	1		1	
Male	56,167	71.0%	0.97 (0.93, 1.00)	0.033	1.15 (1.11, 1.19)	<0.001
Comorbidities						
No	47,182	78.3%	1			
Any	33,899	61.4%	0.44 (0.43, 0.45)	<0.001		
Diabetes mellitus						
No	58,079	73.2%	1		1	
Yes	23,002	66.3%	0.72 (0.70, 0.75)	<0.001	0.93 (0.89, 0.96)	<0.001
Chronic obstructive pulmonary disease						
No	74,449	73.2%	1		1	
Yes	6632	48.8%	0.35 (0.33, 0.37)	<0.001	0.53 (0.50, 0.56)	<0.001
Malignancy						
No	74,017	73.5%	1		1	
Yes	7064	47.3%	0.32 (0.31, 0.34)	<0.001	0.38 (0.36, 0.41)	<0.001
End-stage renal disease						
No	79,172	71.9%	1		1	
Yes	1909	42.9%	0.29 (0.27, 0.32)	<0.001	0.41 (0.37, 0.45)	<0.001
Liver cirrhosis						
No	80,631	71.5%	1		1	
Yes	450	31.1%	0.18 (0.15, 0.22)	<0.001	0.21 (0.17, 0.27)	<0.001
Autoimmune disease						
No	80,262	71.4%	1		1	
Yes	819	59.6%	0.59 (0.51, 0.68)	<0.001	0.69 (0.59, 0.81)	<0.001
Acquired immunodeficiency syndrome						
No	80,632	71.3%	1		1	
Yes	449	53.9%	0.47 (0.39, 0.57)	<0.001	0.37 (0.30, 0.45)	<0.001
Low income						
No	78,213	71.4%	1		1	
Yes	2868	66.5%	0.80 (0.73, 0.86)	<0.001	0.86 (0.79, 0.95)	0.002
Hospital accreditation levels of initial visits						
Medical centers or regional hospitals	43,360	67.0%	1			
Local hospitals or clinics	37,721	76.1%	1.57 (1.52, 1.62)	<0.001	1.25 (1.20, 1.30)	<0.001
Specialties of initial visits						
Pulmonologists or infection specialists	14,102	72.5%	1		1	
Others	66,979	71.0%	0.93 (0.89, 0.97)	<0.001	0.83 (0.79, 0.87)	<0.001
Hospitalisation within 14 days of commencing anti-TB treatment						
No	38,809	82.0%	1		1	
Yes	42,272	61.3%	0.35 (0.34, 0.36)	<0.001	0.62 (0.60, 0.64)	<0.001
Requiring intensive care within 14 days of commencing anti-TB treatment						
No	72,565	75.8%	1		1	
Yes	8516	32.6%	0.16 (0.15, 0.16)	<0.001	0.54 (0.50, 0.59)	<0.001
Invasive ventilatory support within 14 days of commencing anti-TB treatment						
No	73,333	75.7%	1		1	
Yes	7748	28.8%	0.13 (0.12, 0.14)	<0.001	0.39 (0.36, 0.42)	<0.001
Non-invasive ventilatory support within 14 days of commencing anti-TB treatment						
No	79,780	71.9%	1		1	
Yes	1301	30.9%	0.18 (0.16, 0.20)	<0.001	0.75 (0.66, 0.86)	<0.001
Second-line anti-TB treatment ≥14 days						
No	67,190	76.9%	1		1	
Yes	13,891	44.0%	0.24 (0.23, 0.25)	<0.001	0.29 (0.28, 0.31)	<0.001
Delay in anti-TB treatment (per week)					0.992 (0.990, 0.994)	<0.001

*DOTS* directly observed treatment, short course, *OR* odds ratio, *CI* confidence interval

The median treatment delay was 37 (4–107) days. The length of delay increased with age, comorbidities, low-income status, and initially seeking medical help in an urban area (Table 3). Men (coefficient − 6.81 [−7.68 , −5.95]) and performing an MTB–NAAT (coefficient − 2.20 [−3.51, −0.90]) were independently associated with a shorter treatment delay.

**Table 3:** Multivariate linear regression analysis for predictors of length of treatment delay among 81,081 adult patients with pulmonary tuberculosis

		Fulfilling two specific events			Fulfilling three specific events		
	Number	Delay (day)^a^	Coefficient	*P*-value	Delay (day)^a^	Coefficient	*P*-value
Age (years)							
20–34	9595	14 (1–71)	Reference group		4 (0–22)	Reference group	
35–49	13,319	17 (1–77)	2.11 (0.60, 3.62)	0.006	5 (0–30)	2.61 (1.35, 3.86)	<0.001
50–64	17,347	30 (3–95)	8.13 (6.57, 9.61)	<0.001	8 (0–49)	8.61 (7.38, 9.83)	<0.001
65–79	26,897	53 (8–122)	20.0 (18.6, 21.4)	<0.001	20 (0–71)	17.7 (16.6, 18.9)	<0.001
80 and above	13,923	61 (12–128)	23.2 (21.6, 24.7)	<0.001	30 (1–83)	22.7 (21.4, 24.0)	<0.001
Sex							
Female	24,914	42 (7–110)	Reference group		12 (0–56)	Reference group	
Male	56,167	35 (3–105)	−6.81 (−7.68, −5.95)	<0.001	11 (0–58)	−2.50 (−3.21, −1.78)	<0.001
Diabetes mellitus							
No	58,079	35 (4–103)	Reference group		10 (0–54)	Reference group	
Yes	23,002	46 (5–118)	2.97 (2.07, 3.87)	<0.001	14 (0–66)	1.39 (0.65, 2.14)	<0.001
Chronic obstructive pulmonary disease							
No	74,449	33 (3–100)	Reference group		10 (0–52)	Reference group	
Yes	6632	99 (32–154)	30.5 (29.1, 32.0)	<0.001	49 (7–115)	23.7 (22.5, 24.9)	<0.001
Malignancy							
No	74,017	33 (3–101)	Reference group		9 (0–53)	Reference group	
Yes	7064	85 (32–142)	25.7 (24.3, 27.2)	<0.001	44 (7–99)	19.6 (18.4, 20.7)	<0.001
End-stage renal disease							
No	79,172	36 (4–105)	Reference group		11 (0–56)	Reference group	
Yes	1909	92 (35–144)	26.5 (23.8, 29.1)	<0.001	45 (6–108)	22.2 (20.1, 24.4)	<0.001
Liver cirrhosis							
No	80,631	37 (4–107)	Reference group		11 (0–57)	Reference group	
Yes	450	82 (33–138)	29.5 (24.2, 34.7)	<0.001	44 (7–99)	24.1 (19.8, 28.5)	<0.001
Autoimmune disease							
No	80,262	37 (4–107)	Reference group		11 (0–57)	Reference group	
Yes	819	63 (13–133)	14.2 (10.3, 18.1)	<0.001	23 (0–80)	9.59 (6.35, 12.8)	<0.001
Acquired immunodeficiency syndrome							
No	80,632	37 (4–107)	Reference group		11 (0–57)	Reference group	
Yes	449	61 (7–132)	27.9 (22.6, 33.2)	<0.001	19 (0–76)	20.7 (16.4, 25.1)	<0.001
Organ transplantation							
No	80,957	37 (4–107)	Reference group				
Yes	124	83 (42–145)	15.4 (5.34, 25.5)	0.003			
Pneumoconiosis							
No	81,015	37 (4–107)	Reference group		11 (0–57)	Reference group	
Yes	66	109 (63–158)	35.9 (22.2, 49.6)	<0.001	64 (14–118)	27.2 (16.0, 38.4)	<0.001
Low income							
No	78,213	37 (4–106)	Reference group		11 (0–57)	Reference group	
Yes	2868	51 (5–126)	10.1 (7.94, 12.2)	<0.001	14 (0–74)	7.88 (6.13, 9.64)	<0.001
Location of the initial healthcare visits							
Rural area	21,437	36 (4–103)	Reference group		10 (0–52)	Reference group	
Urban area	59,644	38 (4–109)	3.36 (2.46, 4.26)	<0.001	12 (0–60)	4.32 (3.58, 5.07)	<0.001
MTB-NAAT							
No	72,998	38 (4–107)	Reference group		11 (0–58)	Reference group	
Yes	8083	35 (5–104)	−2.20 (−3.51, −0.90)	0.001	13 (0–55)	−1.04 (−2.11, 0.04)	0.058

*Abbreviation*: *AIDS* acquired immunodeficiency syndrome, *COPD* chronic obstructive pulmonary disease, *MTB–NAAT Mycobacterium tuberculosis*–nucleic acid amplification test

^a^Data are expressed as the median (1st–3rd quartiles)

Subpopulation analyses illustrated a stronger impact of performing MTB–NAAT on the treatment delay among the elderly patients (coefficient − 3.97 [−7.09, −0.84]) and smear-negative PTB (coefficient − 4.35 [−6.14, −2.55]) (Additional file 1: Table S4). In the sensitivity analysis adopting a stricter definition of treatment delay, the results were consistent with those in the main scenario (Additional file 1: Tables S5-S6, Table 3).

## Discussion

The present study is the first nationwide report on the outcome of anti-TB treatment in advanced age. It has three crucial findings. First, the incidence rate of PTB is exponentially correlated with the age that elderly adults are the major reservoir of PTB infections in Taiwan. Second, although the anti-TB treatment completion rate has increased following the implementation of directly observed treatment, short course (DOTS) programme in Taiwan since 2006, elder patients with PTB remained to have longer treatment delays and worse outcomes, particularly those with underlying comorbidities. Third, the length of treatment delay is inversely correlated with the treatment completion rate. The treatment delay can be shortened by applying rapid molecular diagnostic tools such as the MTB–NAAT. The extent of benefit is even greater among the elder patients and those with smear-negative PTB. Given the increasing elderly populations worldwide, the findings of the present study can serve as a reference for policies regarding TB care.

According to a World Health Organization report, the TB notification rate increases with age worldwide [4]. As a result of population ageing, the proportion of elder TB patients increased steadily from 1990 to 2015 [17]. The gradual deterioration of the immune system (involving both the host’s capacity to respond to infections and the development of long-term immune memory as age increases, referred to as immunosenescence) may be the major contributor [18, 19]. Other factors, such as malnutrition, poverty, decreased access to health services, comorbidities, and iatrogenic immunosuppression, also contribute to the higher risk of infection in ageing populations [20–22]. However, the correlation between age and PTB incidence has never been calculated, and reports on advanced aged population are currently lacking. This is the first study showing that the risk of PTB not only increases but is exponentially correlated with age (*R* ^2^ = 0.962; Fig. 2).

Because of the high prevalence of underlying comorbidities, anti-TB treatment in elderly patients is frequently complicated by drug–drug interaction and adverse drug reactions, leading to an increased rates of regimen modification and default [8, 23]. Consequently, advanced age increases the mortality rate of TB significantly and eclipses the treatment completion rate [23–27]. In this study, the treatment completion rate among patients ≥65 years old was comparable to the two previous reports (71%–73%) [25, 26]. An even lower treatment completion rate was demonstrated among those with age ≥ 80 years.

Another crucial contributor to poor outcomes in elderly patients with PTB is the delay in anti-TB treatment. The clinical symptoms and radiographic findings of PTB in elderly people tend to be less specific [25, 28, 29]. Extrapulmonary TB including TB meningitis, osteomyelitis or urological involvement is more common with advancing age [3]. Combined with decreased access to health services [30], the atypical manifestations of TB in elder people result in a delay in the diagnosis and treatment [24, 28, 29]. As shown in the present study, prescription of airway medications and antibiotics occurred early in the course prior to chest x ray examination as well as the diagnosis of PTB, suggesting that these cases are already symptomatic and may be infectious in the community and health care system for a long period. Moreover, even when chest radiography is ordered, the duration of treatment delay is still far from negligible, indicating that a high proportion of patients presented with non-diagnostic radiographic findings, particularly in elderly patients. Consistent with previous studies, treatment delay increases mortality rates in patients with PTB [31, 32].

A treatment delay may result from either a delay in seeking health service (patient delay) or failure in establishing diagnosis and starting treatment (provider delay) [6, 33]. In countries with a high TB burden, insufficient patients’ awareness for the TB disease and financial barrier are major contributors for delay in diagnosis [6]. In countries with a low TB burden, the percentage of advanced pulmonary TB with positive sputum smear and cavitary lesions steadily increased due to declining clinicians’ vigilance to the presentations of TB and a lack of efficient diagnostic tools to diagnose TB in its early stage [34, 35]. Because of the built-in shortage of claims data, patient delay cannot be accessed in this study. However, the median of provider delay among patients aged 65–79 years was 32 days longer than that among those aged <65 years (53 vs 21 days). For those aged ≥80 years, the impact can be higher since the treatment delay is longer. In addition to the negative impact on treatment outcome, failure to recognise active PTB cases increases the risk of transmission [36], thus constituting a major hindrance to effective control for TB.

Because ageing is a well-known risk factor for adverse events during anti-TB treatment [8, 23, 37], for safety concerns, physicians are becoming increasingly hesitant to initiate anti-TB treatment unless solid bacteriologic evidence exists. Furthermore, because of the improved accessibility of health services in Taiwan, patients tend to seek medical help while their disease is minimal. This probably explains why an initial medical visit in an urban area is associated with a longer treatment delay than in a rural area. Implementing MTB–NAAT was shown to reduce treatment delay (Table 3), especially among the elderly and smear-negative PTB cases. These findings support the implementation of a rapid molecular assay for PTB diagnosis.

Most patient factors leading to treatment non-adherence can be eliminated with supervision, resulting in an improved treatment completion rate and reduction in unfavourable outcomes [38]. Under Taiwan’s national TB programme, DOTS has been implemented countrywide since 2006. The findings of this study support the continuous government commitment to TB control and the necessity of continuing DOTS programme in Taiwan.

The present study has some limitations. First, because of the built-in shortage of claims data, the results of mycobacterial studies and radiographic findings were unavailable. Second, the disease severity, a critical determinant of patient outcome, was not known. Although hospitalisation and admission to intensive care unit were used as surrogates of disease severity in this study, they may not correlate 100%. Third, the impact of the MTB–NAAT on treatment delay may be confounded by the indication, resulting in an overestimation of it benefits. Lastly and importantly, though the overall delay was calculated by fulfilling two or more events indicating TB onset, they could be due to clinical conditions other than TB. However, it may not be a considerable bias since sensitivity tests showed that the model was consistent across the broader or stricter definitions of delay in treatment.

## Conclusions

The incidence of PTB increased exponentially with age. Ageing is associated with unfavourable outcomes and longer treatment delay, particularly for those with underlying comorbidities. Rapid molecular diagnostic tools can shorten treatment delay and should be integrated in the diagnostic algorithm for PTB, particularly in patients with advanced age.

## Additional file

Additional file 1: Supplemental data for “Pulmonary Tuberculosis in Advanced Age”. The file contains the schematic diagram for delay calculation, the clinical characteristics of patients with pulmonary tuberculosis, treatment delay in pulmonary tuberculosis, medical resource utilisation, subpopulation analyses, and sensitivity analyses (PDF 1066 kb).

## Abbreviations

CT: Computerised tomography
DOTS: Directly observed treatment, short course
ICD-9-CM: International Classification of Diseases, Ninth Revision, Clinical Modification
MTB-NAAT: *M. tuberculosis*–nucleic acid amplification test
NHI: National Health Insurance
NHIRD: National Health Insurance Research Database
PTB: Pulmonary tuberculosis
TB: Tuberculosis
