# Supplementary material for: Treatment delay and fatal outcomes of pulmonary tuberculosis in advanced age: a retrospective nationwide cohort study
Source: BMC Infect Dis. 2017 Jun 24;17:449. doi: 10.1186/s12879-017-2554-y (PMC5483299; doi:10.1186/s12879-017-2554-y)
Supplement: Additional file 1: — Supplemental data for “Pulmonary Tuberculosis in Advanced Age”. The file contains the schematic diagram for delay calculation, the clinical characteristics of patients with pulmonary tuberculosis, treatment delay in pulmonary tuberculosis, medical resource utilisation, subpopulation analyses, and sensitivity analyses (PDF 1066 kb). [file 12879_2017_2554_MOESM1_ESM.pdf]

Fig S1. Schematic diagram for the calculation of delay in anti-tuberculosis treatment (ID, infectious disease; MTB-NAAT, *Mycobacterium tuberculosis*-nucleic acid amplification test)

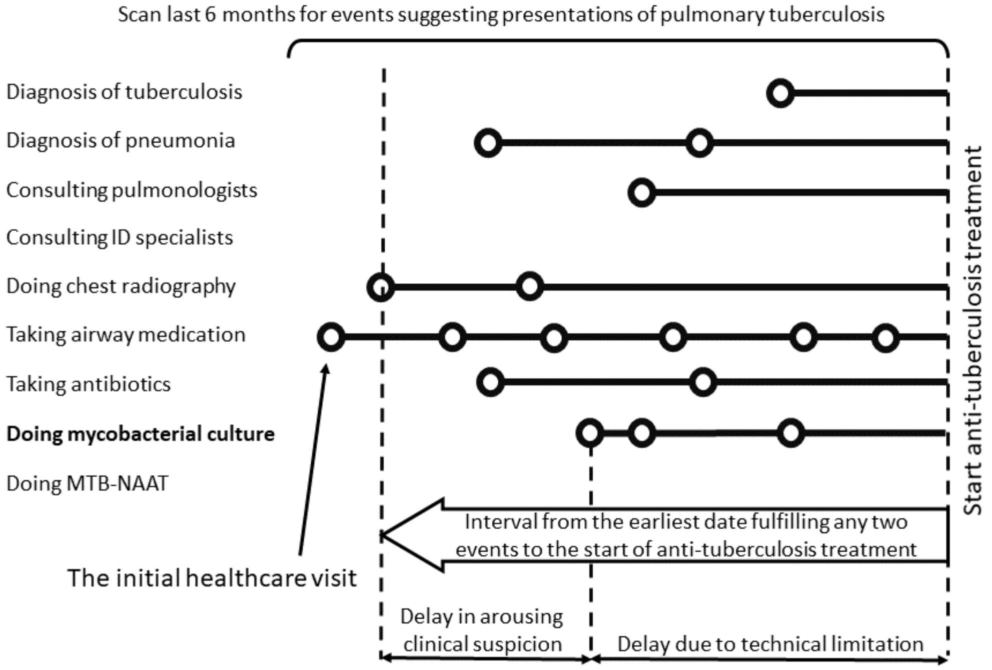

Table S1. Clinical characteristics of the 81081 adult patients with pulmonary tuberculosis diagnosed from 2004 to 2009 stratified by sex

	Female N=24914	Male N=56167	*P*-value
Age (year)	59.8 (40.4–75.8)	66.6 (50.3–77.3)	<0.001
Pre-DOTS era (2004–2005)	8621 (34.6%)	20292 (36.1%)	<0.001
DOTS era (2006–2009)	16293 (65.4%)	35875 (63.9%)	
Any comorbidities	9275 (37.2%)	24624 (43.8%)	<0.001
Diabetes mellitus	6595 (26.5%)	16407 (29.2%)	<0.001
COPD	1301 (5.2%)	5331 (9.5%)	<0.001
Malignancy	1753 (7.0%)	5311 (9.5%)	<0.001
ESRD	839 (3.4%)	1070 (1.9%)	<0.001
Autoimmune disease	538 (2.2%)	281 (0.5%)	<0.001
Liver cirrhosis	106 (0.4%)	344 (0.6%)	0.001
Pneumoconiosis	2 (0.0%)	64 (0.1%)	<0.001
AIDS	39 (0.2%)	410 (0.7%)	<0.001
Transplantation	39 (0.2%)	85 (0.2%)	0.861
Low income status	822 (3.3%)	2046 (3.6%)	0.015
Characteristics of initial healthcare visits			
Hospital accreditation levels			<0.001
Medical centers	3340 (13.8%)	8326 (14.8%)	
Regional hospitals	8744 (35.1%)	22850 (40.7%)	
Local hospitals or clinics	12730 (51.1%)	24991 (44.5%)	
In urban area	18838 (75.6%)	40806 (72.7%)	<0.001
Pulmonologists or infection specialists	3711 (14.9%)	10391 (18.5%)	<0.001
Duration of anti-TB treatment (day)	200 (181–274)	207 (181–278)	<0.001
Treated with isoniazid	184 (126–241)	185 (96–249)	0.431
Treated with rifamycin	183 (149–232)	188 (153–252)	<0.001
Treated with ethambutol	169 (95–211)	172 (99–227)	<0.001
Treated with pyrazinamide	58 (38–78)	61 (37–84)	<0.001
Intensive phase (first 2 months)			
Treated with isoniazid (day)	60 (46–60)	60 (45–60)	0.010

Treated with rifamycin (day)	53 (44–60)	56 (45–60)	<0.001
Treated with ethambutol (day)	56 (43–58)	56 (43–58)	<0.001
Treated with pyrazinamide (day)	48 (28–57)	51 (26–59)	<0.001
Second-line anti-TB drugs ≥14 days	3698 (14.8%)	10193 (18.1%)	<0.001
Diagnostic procedures during the last 2 months before anti-TB treatment			
Bronchoscopy	2535 (10.2%)	5468 (9.7%)	0.053
CT scan	9627 (38.6%)	20960 (37.3%)	<0.001
CT-guided biopsy	450 (1.8%)	945 (1.7%)	0.211
Hospitalisation within 14 days of commencing anti-TB treatment	11933 (47.9%)	30339 (54.0%)	<0.001
Admission to intensive care units	2479 (10.0%)	6037 (10.7%)	0.001
Invasive ventilatory support	2226 (8.9%)	5522 (9.8%)	<0.001
Non-invasive ventilatory support	411 (1.6%)	890 (1.6%)	0.496
Anti-TB treatment outcome at one year			
Completed	17874 (71.7%)	39882 (71.0%)	0.033
Died	3367 (13.5%)	9044 (16.1%)	<0.001
Died within 2 months	1428 (5.7%)	3587 (6.4%)	<0.001

Abbreviations: AIDS, acquired immunodeficiency syndrome; COPD, chronic obstructive pulmonary disease; CT, computerised tomography; DOTS, directly observed treatment, short course; ESRD, end-stage renal disease.

Data are expressed as the median (1st–3rd quartiles) or number (%) as appropriate.

Table S2 Intervals between specified events possibly indicating the onset of pulmonary tuberculosis (TB) and the commencement of anti-TB treatment in different age groups

	No. (%) of patients fulfilling the criteria			Interval from the event to anti-TB treatment (days)		
Age (years)	20–64 (N=40261)	65–79 (N=26897)	80 and above (N=13923)	20–64 (N=40261)	65–79 (N=26987)	80 and above (N=13923)
Clinical diagnosis of tuberculosis	37553 (93.3%)	23481 (87.3%)*	11301 (81.2%)*†	0 (0–1)	0 (0–3)*	0 (0–5)*†
Diagnosis of pneumonia	8365 (20.8%)	8858 (32.9%)*	6616 (47.5%)*†	7 (0–40)	17 (0–62)*	24 (0–69)*†
Consulting pulmonologists	26037 (64.7%)	18 072 (67.2%)*	9583 (68.8%)*†	7 (0–35)	19 (0–76)*	26 (0–93)*†
Consulting ID specialists	3049 (7.6%)	2042 (7.6%)	1227 (8.8%)*†	0 (0–33.5)	8 (0–68)*	21 (0–83)*†
Undergoing chest radiography	33330 (82.8%)	22532 (83.8%)*	11445 (82.2%)†	10 (0–55)	34 (5–95)*	43 (6–104)*†
Taking airway medication	32492 (80.7%)	24038 (89.4%)*	12521 (89.9%)*	79 (17–146)	113 (40–162)*	112 (38–162)*
Taking antibiotics	26922 (66.9%)	20785 (77.3%)*	11830 (85.0%)*†	48 (6–120)	62 (10–130)*	61 (9–129)*
**Receiving mycobacterial culture**	35772 (88.9%)	24478 (91.0%)*	12735 (91.5%)*	3 (0–22)	8 (0–44)*	13 (0–51)*†
Undergoing MTB–NAAT	3753 (9.3%)	2744 (10.2%)*	1586 (11.4%)*†	0 (0–11)	0 (0–15)*	0 (0–16)
Pooled						
Any two of above	40000 (99.4%)	26830 (99.8%)*	13878 (99.7%)*	21 (1–84)	53 (8–122)*	61 (12–128)*†

Any three of above	39010 (96.9%)	26434 (98.3%)[*]	13676 (98.2%)[*]	7 (0–35)	20 (0–71)[*]	30 (1–83)[*†]

Abbreviations: ID, infectious diseases; MTB–NAAT, *Mycobacterium tuberculosis*–nucleic acid amplification test;

Data are expressed as the median (1st–3rd quartiles) or number (%) as appropriate.

[*]*P*-value <0.05 compared against the group with age of 20-64 years. [†]*P*-value <0.05 compared against the group with age of 65-79 years.

Table S3. Medical resource utilisation from the initial healthcare visits to the commencement of anti-tuberculosis treatment among the 81081 adult patients with pulmonary tuberculosis diagnosed from 2004 to 2009

Age (years)	20–64<br>N=40261	65–79<br>N=26897	80 and above<br>N=13923
Number of outpatient visits	12.23±12.78	19.34±17.02*	17.38±15.95*†
Medical centers	2.12±4.35	3.03±5.52*	2.83±5.43*†
Regional hospitals	3.24±5.06	5.84±7.66*	6.25±8.32*†
Local hospitals and clinics	6.87±10.21	10.47±14.03*	8.30±12.36*†
Number of emergency room visits	0.56±1.43	0.84±1.44*	1.10±1.71*†
Number of hospitalisation	0.87±1.13	1.35±1.39*	1.71±1.47*†
Number of chest radiography	1.85±2.29	2.62±3.30*	3.28±4.28*†
Number of mycobacterial culture	2.45±2.22	2.86±2.46*	3.14±2.61*†
Number of MTB-NAAT	0.11±0.41	0.13±0.48*	0.15±0.52*†

Abbreviations: MTB-NAAT, *Mycobacterium tuberculosis* – nucleic acid amplification test.

Data are expressed as the mean ± standard deviation.

*P-value <0.05 compared against the group with age of 20-64 years. †P-value <0.05 compared against the group with age of 65-79 years.

Table S4. Subpopulation analyses for the impact of *Mycobacterium tuberculosis*–nucleic acid amplification tests on shortening treatment delay in multivariate linear regression analyses

Subpopulation	Coefficient	*P* value
All patients	-2.20 (-3.51, -0.90)	0.001
Age ≥65 years	-3.23 (-5.10, -1.36)	0.001
Age ≥80 years	-3.97 (-7.09, -0.84)	0.013
Smear-negative	-4.35 (-6.14, -2.55)	<0.001

Table S5. Sensitivity analyses for different definitions of delay in treatment by multivariate logistic regression for predictors of complete anti-tuberculosis treatment within 12 months

Definitions for calculating delay	Adjusted odds ratio*	*p*-value
Fulfilling ≥2 specific events (per week)	0.992 (0.990, 0994)	<0.001
Fulfilling ≥3 specific events (per week)	0.986 (0.984, 0.989)	<0.001
Fulfilling ≥4 specific events (per week)	0.983 (0.980, 0.986)	<0.001

*Adjusted for age, sex, implementation of directly observed treatment, short course, diabetes mellitus, chronic obstructive pulmonary disease, malignancy, end-stage renal disease, liver cirrhosis, acquired immunodeficiency syndrome, low income, hospital accreditation levels of initial visits, specialties of initial visits, baseline hospitalization, intensive care, invasive ventilatory support, non-invasive ventilatory support, and second-line anti-tuberculosis treatment.

Table S6. Sub-population analyses for the impact of *Mycobacterium tuberculosis* – nucleic acid amplification test to shorten the delay in anti-tuberculosis treatment in multivariate linear regression analysis by adopting a strict definition for delay in anti-TB treatment.

Sub-population	Coefficient	*p*-value
All patients	-1.04 (-2.11, 0.04)	0.058
Age ≥ 65 years	-2.47 (-4.08, -0.86)	0.003
Age ≥ 80 years	-3.65 (-6.40, -0.90)	0.009
Smear-negative	-5.16 (-8.09, -2.23)	0.001
